# Effectiveness of Prenatal Intervention on the Outcome of Diseases That Have a Postnatal Urological Impact

**DOI:** 10.3389/fped.2019.00118

**Published:** 2019-04-02

**Authors:** Beatiz Bañuelos Marco, Ricardo González, Barbara Ludwikowski, Anja Lingnau

**Affiliations:** ^1^Department of Urology, Charité Medical University of Berlin, Berlin, Germany; ^2^Pediatric Surgery and Urology, Kinder- und Jugendkrankenhaus AUF DER BULT, Hanover, Germany

**Keywords:** LUTO, congenital adrenal hyperplasia (CAH), vesicoamniotic shunt, myelomeningocele, fetal therapy, posterior urethral valves

## Abstract

We reviewed the literature addressing outcomes of fetal intervention of conditions that require post-natal urological management including lower urinary tract obstruction, hydrometrocolpos, congenital adrenal hyperplasia, and myelomeningocele. Despite several decades of fetal intervention for these conditions, benefits remain elusive in part because of the enormous difficulty of conducting prospective randomized studies. In this review, we reached the following conclusions:

1. Prenatal intervention in lower urinary tract obstruction (LUTO) improves survival in the worst cases by improving pulmonary function and it may be advantageous for renal function, particularly in patients with adverse urine parameters.
2. Prenatal treatment of female fetuses at risk of having congenital adrenal hyperplasia (CAH) reduces virilization. Non-invasive fetal DNA analysis allows earlier diagnosis, reducing the risk of treating with dexamethasone males and non-affected fetuses.
3. Fetal treatment of myelomeningocele (MMC) is gaining momentum. Prospective studies including standardized urologic evaluation will determine if the encouraging results reported by some hold on the long term follow-up.

## Introduction

The purpose of this minireview is to present current knowledge of the effectiveness and impact of prenatal intervention in fetuses with congenital anomalies which have urological consequences in postnatal life. Prenatal detection of genito-urinary tract anomalies has been possible for about 50 years and prenatal surgical interventions for lower urinary tract obstruction (LUTO) date back to the 1980s (1). Significant technical improvements in ultrasonography (US) technology and the addition of fetal MRI have led to significant refinements in prenatal diagnosis. In addition, fetal genetic testing has allowed the prenatal diagnosis of many diseases, among them congenital adrenal hyperplasia (CAH) the prenatal treatment of which impacts postnatal management and outcome. Nevertheless, prenatal treatment of many conditions diagnosed prenatally remains controversial. Here we briefly summarize the current literature regarding prenatal management of fetal lower urinary obstruction, hydronephrosis, hydrocolpos, congenital adrenal hyperplasia, and myelomeningocele.

## Method

Literature review searching PubMed, Cochrane, and Embase databases using pertinent key words for each subject of this review. Articles from the last 10 years were preferentially included for this review. Older articles were included if considered important.

## Results

### Lower Urinary Tract Obstruction (LUTO)

Fetal LUTO refers to the combination of bilateral hydroureteronephrosis and a persistently distended bladder (2). It occurs in 2.2/10,000 pregnancies (2). Fetal LUTO in the second trimester of gestation can lead to postnatal renal failure as well as early respiratory insufficiency due to pulmonary hypoplasia resulting from diminished or absent amniotic fluid. The principal causes of LUTO are posterior urethral valves, Eagle Barret syndrome (prune belly syndrome or PBS) and urethral atresia (3). The mortality of fetus with bilateral hydronephrosis, a distended bladder and oligo- or anhydramnios in the second trimester of life approaches 100% without intervention. End stage renal insufficiency will affect a high proportion of surviving fetuses leading to dialysis and the need of a transplant at the age of 5 years (4).

In the course of the last 4 decades studies tend to demonstrate an early survival advantage by placing a percutaneous shunt between the bladder and the amniotic cavity (VAS) (5). Although VAS is the most common treatment to restore the amniotic fluid volume and alleviate the obstruction, the rate of complications is still very high, 40% (4), and the long term renal function remains uncertain. Anecdotal cases of survival and even good renal function in cases of early bladder decompression are published (6, 7) but prospective series are rare. Survival is improved on the perinatal period, but at 6 months and 2 years follow up there is no evidence of neither a better survival rate nor renal function. Oligohydramnios on the 16–24 weeks of pregnancy can cause irreversible pulmonary hypoplasia (4, 8).

A recent publication by Ruano et al. proposed a new, standardized approach to the selection of patients with LUTO based on multiple criteria including amniotic fluid levels, renal appearance, and fetal urinary electrolyte assessment. The biochemistry and ultrasound characteristics of the fetal kidneys are important to estimate fetal renal function (9).

However, the evaluation of the fetal renal function is difficult since sonographic parameters are not sensible enough and according to some authors biochemical parameters have poor clinical accuracy in the selection of those patients that could benefit from the procedure (10). However, non-favorable results in the biochemistry analysis of the fetal urine correlate with irreversible histological changes in the fetal kidney and aid in identifying fetuses which will not benefit from intervention (11). In a recent publication including 89 patients with prenatally detected posterior urethral valves followed, 20% of the patients were followed for at least 20 years. The authors report that postnatal glomerular filtration rate was >60 mL/min/1.73 m^2^ in 67.4% of the cases and <30 mL/min/1.73 m^2^ in 17%. A combination of β2-microglobulin and chloride had the best prognostic value (93% sensitivity and 71% specificity). They concluded that fetal urine parameters predicted long-term postnatal renal function (12) confirming earlier more preliminary observations (13).

### Posterior Urethral Valves (PUV) Are the Most Common Cause of LUTO

More than one third of the boys with PUV have a lifetime risk for end-stage renal disease (14). Conversely, PUV accounts for 18% of the pediatric end-stage renal disease. In fetal ultrasonography in the second trimester, the keyhole sign (appearance of a distended bladder and prostatic urethra is the most common sign of PUV (1–3).

Urethral atresia (UA) is seen in 0.3/10,000 births. In the absence of spontaneous decompression of the bladder or prenatal intervention, the condition is invariably fatal.

Prune Belly Syndrome (PBS), also known as the triad syndrome or Eagle Barret syndrome in seen in 1/30,000–40,000. The association of PBS with urethral obstruction (which may no longer be present at the time of diagnosis) is no longer disputed (15, 16).

### Prenatal Treatment of LUTO

Prenatal treatment of LUTO dates back more than 3 decades (17). From initial open vesicostomy, treatment have evolved to percutaneous placement of vesicoamniotic shunts to fetoscopic surgery. While open bladder decompression has been abandoned, debate continues regarding the superiority of shunting vs. a fetoscopic approach.

### Vesicoamniotic Shunt (VAS)

In 1986, International Fetal Surgery Registry reported 41% survival after VAS placement (18). With current techniques and patient selection survival is now approaching 70% in the first year (19).

Nassr et al. published meteanalysis which included 9 articles of fetal VAS placement with a follow up to 2 years and better defined diagnosis of LUTO (4). One-hundred-twelve fetuses were shunted and 134 fetuses treated conservatively. Shunted fetuses had statistically better survival in the first 6 months of life (57% VAS vs. 39% observed), but it didn't impact survival between 6 and 12 months. Also there wasn't any evidence that VAS placement improved postnatal renal function. However, it should be noted that the effect of VAS placement was less evident in those patient with better prognosis based on favorable fetal urinary chemistry. In contrast those with poor prognostic features benefitted from VAS placement. Ruano et al. also reported better survival after shunting (8). Historically, Freedman et al. reported series of 55 fetuses with LUTO who received VAS and concluded that: “When evaluated by specific diagnosis, intervention appears to provide outcomes in these high risk fetuses that are comparable to those for disease detected post-natally” (3). A recent meta-analysis also suggests that VAS may improve renal function (20). Unfortunately, until today the rate of complications of VAS reaches almost a 40% including shunt migration, obstruction, and displacement (9).

### Fetal Cystoscopy

Fetal cystoscopy was reported to aid in making an accurate diagnosis at the time of prenatal intervention (21). Cystoscopy permits simultaneous evaluation of the bladder and urethra and even laser ablation of posterior urethral valves (22–25) but after more than 20 years of attempting such treatment the risk of creating fistulas remains high.

In the retrospective study from Ruano et al. (9, 26), 111 fetuses with severe LUTO were evaluated between 1990 and 2013. In 34 fetuses cystoscopy was performed, in 16 VAS was placed and nothing was performed in 16. The overall survival was higher in the group of patients who underwent any kind of treatment; being 43.8% in the VAS group, 38.2% in the fetal cystoscopy group and 19.7% in the group who did not undergo treatment. The 6 months survival rate show no statistically difference between the fetal cystoscopy group and the VAS group. The evaluation of the renal function showed that compared with no treatment fetal cystoscopy significantly improved the renal function, an improvement that could not be demonstrated on the VAS group. After performing multivariate analysis and adjusting for all variables including gestational age at diagnosis of LUTO, type of fetal intervention given, postnatal diagnosis of the cause of LUTO and fetal center location, as well as considering termination of pregnancy, fetal cystoscopy was associated with a significant improvement in the 6 months survival rate and a not significant trend toward normal renal function. Fetal VAS did not show a significant improvement in renal function. Also in this study, complications of those patients undergoing cystoscopy and intervention was high: fistula in 8.8%, and recurrence of LUTO 5.9% which leaded to preterm delivery and death after birth. Complications of VAS, mainly obstruction or migration, occurred in 31.3%.

### Other Urinary Tract Anomalies

In cases of mild upper tract dilatation there is a 11.9% risk of pathological findings, and 1/10 fetuses presenting mild hydronephrosis has a serious condition (27). Ureteroceles may cause bladder outlet obstruction with similar consequences as other cases of LUO. Prenatal detection and fetoscopic treatment has been reported (28, 29).

Thus, prenatal intervention in LUTO improves survival in the worst cases by improving pulmonary function and it may be advantageous for renal function, particularly in patients with adverse urine parameters.

### Hydrocolpos and Cloaca

A retrovesical cystic mass in a female fetus usually represent a Mullerian structure filled with fluid. Mallman et al. reported 20 cases which illustrate the spectrum of differential diagnoses, associated malformations and possible course of action (30). Most of the cases were detected in the late ultrasound screening, and only one in the second semester screening (median gestational age at diagnosis 30 weeks). Intra-abdominal cystic masses present a difficult differential diagnosis which may include hydronephrosis, fetal ovarian cysts, anterior cystic teratoma, cloacal malformation, intra-abdominal teratoma, anterior meningocele, and hydrocolpos. Most of the fetuses presenting hydrocolpos will prove to have a severe malformation. Final diagnoses in this series included anorectal malformation in 13, anal atresia, cloacal malformation, cloacal exstrophy intra-abdominal teratoma, vaginal atresia or duplication, persistent urogenital sinus or cardiac malformations. Two of twenty parents opted for termination of pregnancy, three suffered intrauterine fetal death. Fifteen were born at median gestational age of 35 weeks and underwent postnatal reconstructive procedures. Of interest is that the association with ascites indicated a severe associated malformation such as a cloacal malformation (31). Persistent urogenital sinus with hydrometrocolpos is a potentially treatable malformation but the fetal survival depends on the other associated anomalies and the time of prenatal diagnosis. Prenatal decompression may be used in selected cases (32).

Prenatal detection of hydrometrocolpos rarely leads to prenatal intervention but allows improved family counseling.

### Congenital Adrenal Hyperplasia (CAH)

Congenital adrenal hyperplasia due to 21-hydroxylase deficiency is an autosomal recessive disorder caused by mutations in the CYP21A2 gene. The incidence of the classic form varies between 1/15,000 and 1/24,000, more common in Asians and Caucasians than in Africans. In the classic form synthesis of cortisol and aldosterone are impaired which may result in adrenal insufficiency and salt wasting and in females, virilized genitalia (33).

Genetic studies reveal a mutation in the CYP21A2 gene. Although numerous mutations have been reported, 10 are found in the majority of cases. Prenatal genetic studies allow early detection of the mutation in subsequent pregnancies.

Prenatal diagnosis is recommended in pregnancies when both parents are carriers of the disorder, one parent has a clinical manifestations of CAH or there an affected family member (34). German guidelines recommend prenatal diagnosis in the following cases: (1) Families with one affected child (index case) with classic CAH (CYP21A2); (2) Known parental heterozygosity for classic CAH (no index case) (3) New relationship of a parent of a child with classic CAH if the new partner is known to be a carrier for classic CAH; (4) Homozygosity or compound heterozygosity for classic CAH of one parent when the other parent is a heterozygous gene carrier for classic CAH (35).

As recently as 2015 prenatal treatment of fetuses at risk of virilization was considered experimental in Germany. Part of the reason for this reservation was that for the treatment with dexamethasone to be effective, it had to be initiated before the diagnosis could be confirmed by chorionic villous sampling which expose non-affected fetuses to unnecessary treatment. New developments in the diagnosis by non-invasive methods performing genetic testing of fetal DNA extracted from maternal blood might make the treatment more acceptable (36).

The aim of the prenatal treatment is to avoid virilization of the female fetus. The treatment with dexamethasone was introduced in 1978 by Forest in France and in 1986 in the U.S. Although highly effective if used consistently before the 8 week of those pregnancies at risk, it is not absent of controversies due to animal studies showing effects on brain development (37) but the issue remains controversial (38). With older invasive diagnostic methods (chorionic villus sampling), the sex of the child is determined between 12th and 14th weeks, treatment is suspended if the child is a male or a non-affected female which meant unnecessarily treating 7 of 8 fetuses (37). Non-invasive DNA analysis may overcome these objections (36, 39).

Nevertheless, other potential adverse effects on the cardiovascular, renal and metabolic functions are likely but not yet well-understood (40).

Prenatal treatment of female fetuses at risk of having CAH reduces virilization. Non-invasive fetal DNA analysis allow earlier diagnosis, reducing the risk of treating with dexamethasone males and non-affected fetuses.

### Myelomeningocele

Myelomeningocele (MMC) is a life altering birth defect, which results from an incomplete closure of the neural tube during the fourth week of gestation. It affects 5 of 10,000 births (41). Prenatal closure of the myelomeningocele aims at reducing the noxious effect of the amniotic fluid on the exposed spinal cord (42). Fetal repair of has been done by open surgery, fetoscopic surgery, or a combination of both.

The MOMS trial (43) was a multicenter study comprising 183 fetuses of which 158 were evaluated at 12 month of age that demonstrated that, within the period of the follow-up the incidence of ventriculo-peritoneal shunt placement were reduced from 82% in the group operated after birth to 40% in the prenatally operated group. Other potential benefits of prenatal closure included better mental development and motor function. Disadvantages of fetal closure included an increased risk of preterm delivery and uterine rupture at delivery.

Since first MMC closure 1998 (44), nine reports of urological outcomes on 291 cases have been published worldwide (45–52). Most reports that compared early bladder function of MMC patients treated pre- or post-natally failed to show nay advantages. However, there are two encouraging reports. Carr, based on interviews with 54 patients or families who had prenatal closure of MMC, reported that 10 children (18.5%) were toilet-trained, two patients had stool continence, and one was continent of urine but required bowel management, suggesting better outcomes than expected with postnatal closure (53).

Horst et al. reported urodynamic outcomes in 8 of 30 children with 2 year follow-up whose MMC was closed prenatally and compared them with a group with conventional treatment (51). Although they report that bladder function was normal in half of the patients. However, given the difficulties of evaluating bladder function before toilet training the results must be interpreted with caution. Bladder wall thickness was significantly more frequent in prenatally treated patients. This may be the result of better neurological outcome or sphincter denervation as reported by others (47).

Fetal treatment of MMC is gaining momentum. Prospective studies including standardized urologic evaluation will determine if the encouraging results reported by some hold on the long term follow-up.

## Conclusions

1. Prenatal intervention in LUTO improves survival in the worst cases by improving pulmonary function and it may be advantageous for renal function, particularly in patients with adverse urine parameters. Development of better instrumentation would improve the success of fetal cystoscopy and reduce complications.
2. Prenatal treatment of female fetuses at risk of having CAH reduces virilization. Non-invasive fetal DNA analysis allow earlier diagnosis, reducing the risk of treating with dexamethasone males and non-affected fetuses.
3. Fetal treatment of MMC is gaining momentum. Prospective studies including standardized urologic evaluation will determine if the encouraging results reported by some hold on the long term follow-up.

## Author Contributions

All authors contributed to the conception of this review. BB and RG conducted the review of the literature and wrote the manuscript. AL and BL contributed substantially to the preparation of the manuscript.

### Conflict of Interest Statement

The authors declare that the research was conducted in the absence of any commercial or financial relationships that could be construed as a potential conflict of interest.
